# Do Circulating Redox Biomarkers Have Diagnostic Significance in Alcohol-Intoxicated People?

**DOI:** 10.3390/ijms231911808

**Published:** 2022-10-05

**Authors:** Mateusz Maciejczyk, Iwona Ptaszyńska-Sarosiek, Anna Niemcunowicz-Janica, Michał Szeremeta, Napoleon Waszkiewicz, Agnieszka Kułak-Bejda, Urszula Cwalina, Miłosz Nesterowicz, Anna Zalewska

**Affiliations:** 1 Department of Hygiene, Epidemiology, and Ergonomics, Medical University of Bialystok, 15-089 Bialystok, Poland; 2 Department of Forensic Medicine, Medical University of Bialystok, 15-089 Bialystok, Poland; 3 Department of Psychiatry, Medical University of Bialystok, 15-089 Bialystok, Poland; 4 Department of Statistics and Medical Informatics, Medical University of Bialystok, 15-089 Bialystok, Poland; 5 Students Scientific Club “Biochemistry of Civilization Diseases” at the Department of Hygiene, Epidemiology and Ergonomics, Medical University of Bialystok, 15-089 Bialystok, Poland; 6 Department of Restorative Dentistry and Experimental Dentistry Laboratory, Medical University of Bialystok, 15-089 Bialystok, Poland

**Keywords:** alcohol poisoning, alcohol toxicity, oxidative stress, redox biomarkers, circulating biomarkers

## Abstract

The toxic properties of ethanol are inextricably linked to oxidative stress. Despite many reports on the effects of alcohol dependence on blood redox homeostasis, there are no data on the oxidative stress profile in alcohol-poisoned cases. There are also no data on the diagnostic usefulness of redox biomarkers determined post-mortem in various biological fluids. This work investigates the utility of enzymatic and non-enzymatic antioxidant barrier, redox status, and oxidative/nitrosative stress biomarkers in different biological fluids (such as blood, urine, vitreous humor, and cerebrospinal fluid) in the post-mortem study of patients with acute alcohol intoxication. The study group included those who died due to acute ethanol intoxication (*n* = 22). The research showed a significant increase in glutathione peroxidase activity, total antioxidant status, ferric reducing antioxidant power, and tryptophan concentration only in the study group’s urine compared to the control. In other circulating fluids, both antioxidant enzyme activities and glycoxidation product concentrations were not significantly different in individuals who died of alcohol overdose compared with those who died suddenly. We also did not observe a connection between oxidation–reduction balance and the amount of alcohol consumed before death. These unexpected observations may be caused by irreversible post-mortem changes occurring at the cellular level due to autolysis and putrefaction. In summary, the use of circulating body fluids to assess redox homeostasis is limited in the post-mortem analysis. Our results indicate the increased stability of urine collected post mortem compared to other circulating bioliquids. Further studies are needed to assess the intensity of oxidative and carbonyl stress in ethanol-damaged organs and the effects of post-mortem processes on cellular redox balance.

## 1. Introduction

Alcohol is one of the most widely misused stimulants in developed and developing countries [1]. Chronic alcohol intake is associated with many pathophysiological changes, including central nervous system (CNS) disorders, cancers, pancreatic disease, liver cirrhosis, diabetes, osteoporosis, arthritis, kidney disease, gastrointestinal disorders, immune dysfunction, hypertension, and cardiomyopathy, as well as heart failure [2,3,4,5,6,7,8,9,10]. Annually, alcohol abuse leads to about 3.3 million deaths, accounting for 5.9% worldwide deaths [11]. Nevertheless, single alcohol consumption may also cause sudden death. It was shown that a high amount of alcohol inhibits CNS function, causes respiratory depression, and may lead to death by asphyxia [12]. However, autopsy studies of acute alcohol intoxication cases lacked specific anatomical–pathological findings compared with other forensic patients. It is not surprising that differences in the proteomic, genomic, and metabolic profiles are continually being sought in individuals who died of alcohol overdose [13].

Oxidative stress is the imbalance between pro-oxidants and antioxidants, manifested by an accumulation of oxidized molecules in the tissues [14]. It is a situation when the steady-state reactive oxygen species (ROS) level is transiently or chronically enhanced, disturbing cellular metabolism and damaging cellular constituents [15]. A significant role in preventing oxidative stress is attributed to enzymatic and non-enzymatic antioxidants, which inhibit the formation of free radicals and participate in their conversion into nonreactive derivatives [16]. The antioxidant enzymes are characterized by a more excellent selectivity of action, while the remaining antioxidants scavenge free radicals, interrupting the oxidation reaction in the cell [17].

There is accumulating evidence pointing to oxidative stress as a mechanism of alcohol toxicity [18,19,20]. In the liver, ethanol is primarily oxidized to the reactive intermediate acetaldehyde and then to acetate by the cytosolic alcohol dehydrogenase (ADH) and the mitochondrial aldehyde dehydrogenase (ALDH). Acetaldehyde can readily react with proteins’ amino, hydroxyl, and thiol groups, altering their function and enzymatic activity [21,22]. Moreover, alcohol metabolites can indirectly stimulate liver injury by changing the gut microbiome, accompanied by impaired gut barrier function, increased leaky gut, and the translocation of intestinal bacteria with high plasma endotoxin levels (e.g., lipopolysaccharide) [23,24]. Excessive amounts of alcohol can also be oxidized to acetaldehyde by ethanol-inducible cytochrome P450-2E1 (CYP2E1), which generates superoxide anion and hydrogen peroxide [25]. It is not surprising that changes in antioxidant enzyme activity and increased oxidation of proteins, lipids, and DNA have been demonstrated in acute and chronic alcohol intoxication [26,27,28,29,30]. Despite many reports of redox homeostasis in alcohol-dependent individuals, there are no data on oxidative stress profile in people poisoned by alcohol. In addition, there are no data on the diagnostic utility of redox biomarkers assessed post mortem in various biological fluids. Although oxidation products of biomolecules are much more durable than free radicals, it is not known whether their post-mortem assessment has a diagnostic value [31,32]. Various decomposition processes (autolysis and putrefaction) occur after death; however, their effect on the redox homeostasis of body fluids is still unclear [33]. The study aimed to assess the enzymatic and non-enzymatic antioxidant barrier, redox status, and oxidative/nitrosative stress biomarkers in the blood, urine, cerebrospinal fluid, and vitreous humor of people who died intoxicated with ethanol.

## 2. Results

The authors measured levels of ethyl alcohol in blood, urine, and other materials collected during the autopsy. The study group included individuals who showed very high levels of alcohol in the blood and the urine: in serum 4–4.5‰ and urine 4–6.1‰. The presence of acetaldehyde was also qualitatively confirmed in the samples tested. The deceased in the control group were sober. The height of individuals in the study group was 165–189 cm. In the control group, it was 167–184 cm.

### 2.1. Antioxidant Assays

**Figure 1:**
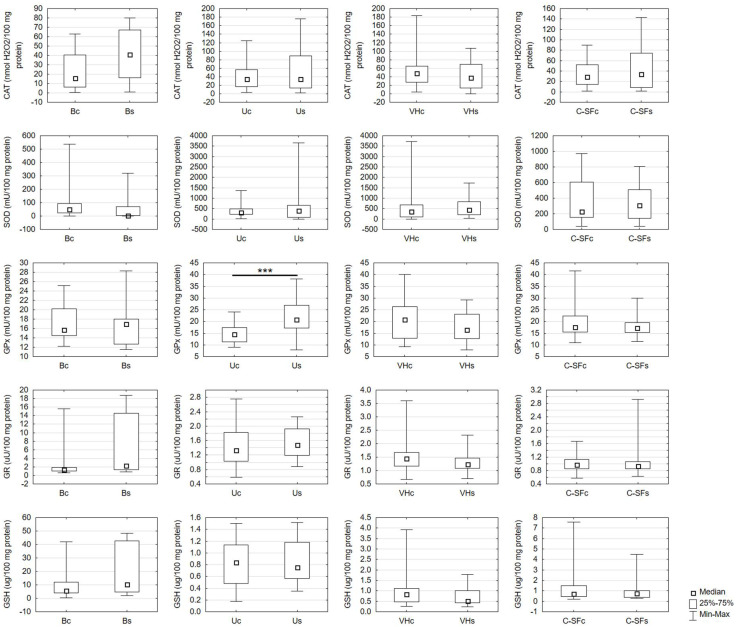
Antioxidant assays in the control and the study group in biological fluids. Bc, blood of the control group; Bs, blood of the study group; CAT, catalase activity; C-SFc, cerebrospinal fluid in the control group; C-SFs, cerebrospinal fluid in the study group; GPx, glutathione peroxidase activity; GR, glutathione reductase activity; GSH, reduced glutathione level; SOD, superoxide dismutase-1 activity; Uc, urine of the control group; Us, urine of the study group; VHc, vitreous humor of the control group; VHs, vitreous humor of the study group; *** *p* < 0.001 vs. control.

GPx activity was effectively increased only in the urine of the study group compared to the control group. The activity of CAT, SOD-1, and GR and the level of GSH were not significantly different between the study and the control groups in any biological fluid (Figure 1).

### 2.2. Redox Status Assays

**Figure 2:**
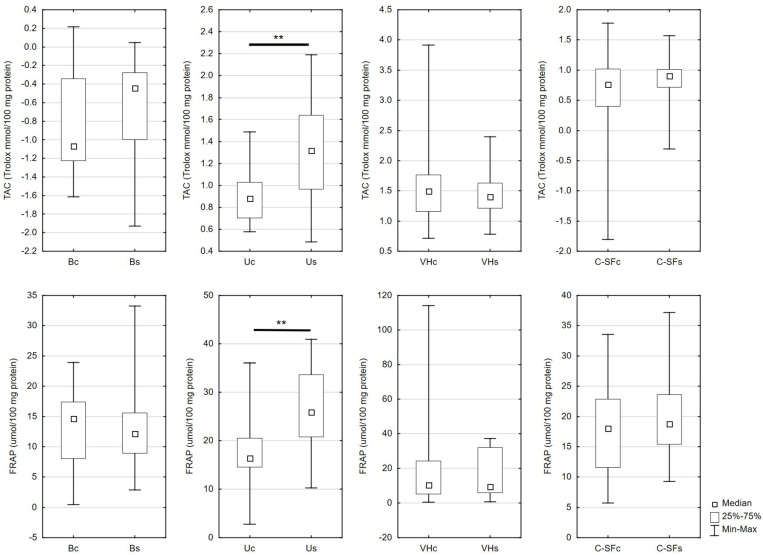
Redox status assays in the control and the study group in biological fluids. Bc, blood of the control group; Bs, blood of the study group; C-SFc, cerebrospinal fluid in the control group; C-SFs, cerebrospinal fluid in the study group; FRAP, ferric reducing ability of plasma level; TAC, total antioxidant capacity level; Uc, urine of the control group; Us, urine of the study group; VHc, vitreous humor of the control group; VHs, vitreous humor of the study group; ** *p* < 0.01 vs. control.

Urinary levels of TAC and FRAP were considerably higher in the study than in the control group. These parameters were not significantly different in blood, vitreous body, and cerebrospinal fluid (Figure 2).

### 2.3. Oxidative Damage and Nitric Oxide Assays

**Figure 3:**
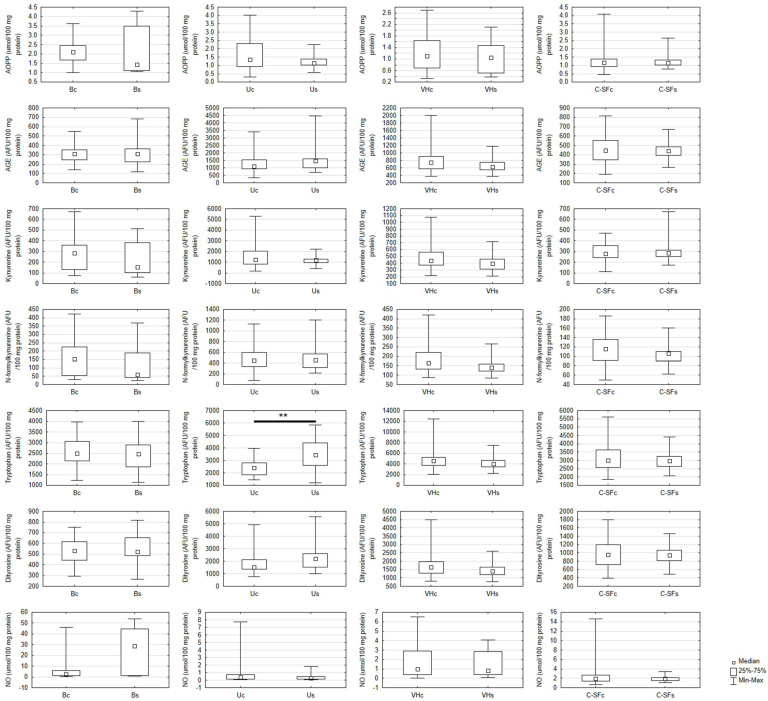
Oxidative damage and nitric oxide assays in the control and the study group in biological fluids. AGE, advanced glycation end products level; AOPP, advanced oxidation protein products level; Bc, blood of the control group; Bs, blood of the study group; C-SFc, cerebrospinal fluid in the control group; C-SFs, cerebrospinal fluid in the study group; NO, nitric oxide level; Uc, urine of the control group; Us, urine of the study group; VHc, vitreous humor of the control group; VHs, vitreous humor of the study group; ** *p* < 0.01 vs. control.

Tryptophan concentration was efficiently enhanced only in the urine of the study group in comparison to the control group. There were no significant differences in other oxidative damage markers (AOPP, AGE, dityrosine, kynurenine, and N-formylkynurenine content) and NO level between the study group and the control group in any body fluid (Figure 3).

### 2.4. Protein Assay

**Figure 4:**
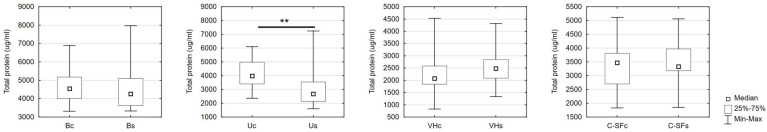
Protein assay in the control and the study group in biological fluids. Bc, blood of the control group; Bs, blood of the study group; C-SFc, cerebrospinal fluid in the control group; C-SFs, cerebrospinal fluid in the study group; Uc, urine of the control group; Us, urine of the study group; VHc, vitreous humor of the control group; VHs, vitreous humor of the study group; ** *p* < 0.01 vs. control.

Total protein content was significantly reduced only in the urine of the study when compared to the control group (Figure 4).

### 2.5. Correlations

We did not show any significant correlations between redox biomarkers in different biological fluids and alcohol content.

### 2.6. Multifactorial Linear Regression

Regression analysis was used to assess the effect on biomarker concentrations of variables such as alcohol concentration and cause of death. The models were further adjusted for gender and age. In general, there was no association between the factors studied and the level of biomarker concentrations. The exceptions were measurements made in urine. However, it is worth noting that the coefficients of determination for the following models are very low, which indicates their low significance (Table S1).

## 3. Discussion

Our study is the first to compare blood, urine, vitreous, and cerebrospinal fluid biomarkers of oxidative stress in individuals who died of alcohol overdose. We have shown that post-mortem assessment of oxidative stress has limited diagnostic value in circulating bioliquids.

The toxic effects of ethanol are inextricably linked to oxidative stress [1]. Acetaldehyde and acetic acid formed by the ADH and ALDH enzymes are mainly responsible for alcohol poisoning [2]. In alcohol oxidation, cellular stores of glutathione and NAD are depleted [3], inhibiting the breakdown of lactic acid and resulting in metabolic acidosis [4]. The reduction of glutathione reserves may also be associated with the overproduction of acetaldehyde, which, like other toxic compounds, is removed from the cell by glutathione S-transferase [5,6]. Additionally, acetaldehyde and acetic acid form adducts with hemoglobin or albumin and react with the amino groups of proteins to form Schiff bases [7,8]. Therefore, both ethanol and its intermediates have pro-oxidant solid properties [9]. Chronic alcohol use also induces the MEOS system, a source of hydrogen peroxide, superoxide anion, and hydroxyl radical, which converts to more toxic hydroxyethyl radicals [10,11]. Due to their long half-life, hydroxyethyl radicals predominantly contribute to the oxidation of proteins, lipids, and DNA in chronic alcoholic drinkers [12]. Ethanol is a highly lipophilic substance that quickly penetrates all tissues and passes through the blood–brain barrier [13]. Both acute and chronic alcohol abuse leads to disturbances in redox homeostasis manifested as exhaustion of antioxidant systems (e.g., glutathione, vitamin E, and enzymatic antioxidants) and enhanced oxidative damage in several body organs [14,15,16,17].

The assessment of oxidative stress intensity determines the degree of cellular injury and often the severity of redox-mediated diseases [18]. Redox biomarkers are postulated in the diagnosis of metabolic, autoimmune, neurodegenerative, or neoplastic diseases, given the critical role of oxidative stress in their pathogenesis [19,20,21,22,23,24,25]. Although direct analysis of ROS production is a challenging task, products of protein, lipid, and DNA oxidation are most commonly used to evaluate redox status [26]. Oxidative modifications products are much more durable than free radicals and thus easier to analyze [27]; however, little is still known about various biological fluids’ redox profiles, such as urine, vitreous, and cerebrospinal fluid. There are also no data on the usefulness of circulating redox biomarkers collected after death [28]. Considering the ROS-mediated alcohol toxicity, we postulated that assessment of oxidative stress in body fluids could estimate the effects of alcohol exposure in lethally intoxicated individuals.

Nevertheless, in our study, both the activity of antioxidant enzymes and the concentration of protein oxidation/glycoxidation products were not significantly different in subjects who died of alcohol overdose compared to those who died suddenly. We also observed no relationship between the oxido-reductive balance and the amount of alcohol consumed before death. These surprising observations may be explained by post-mortem changes, which are manifestations of the final and irreversible death at the cellular level [29]. Post-mortem decomposition includes autolysis involving the breakdown of tissues by the body’s enzymes and putrefaction caused by putrefactive bacteria [30]. Neurons undergo autolysis most rapidly (even a few minutes after death), while cells of parenchymal and enzyme-rich organs (e.g., liver, kidney, pancreas, and intestines) suffer autolysis later [31]. Hemolysis of the blood also occurs very quickly (as early as 2–3 h after death), which is not insignificant in laboratory diagnostics and forensics [32].

On the one hand, the release of analytes contained inside the cells causes an increase in their concentration in plasma. However, on the other hand, free hemoglobin and other blood components may have the opposite effect. The released constituents may interfere with the assay reagents and cause false-negative results [33,34,35]. Therefore, the post-mortem evaluation of the blood redox profile may lead to erroneous results. Our study found hemolysis in all blood samples, which disqualifies it for evaluating circulating redox biomarkers. However, the results obtained for CSF, vitreous, or urine may also be questionable. The relatively high protein content may evidence the decomposition of the analyzed body fluids, and, as a result of cell membrane injury, the protein may have been released from surrounding tissues [36]. Not surprisingly, we noted high protein contents in urine and CSF, which physiologically occurs at low levels [37]. It should be noted that blood, urine, cerebrospinal fluid, or vitreous is commonly used in forensic medicine, mainly for toxicological analyses [38]. Our results indicate the limited use of body fluids for assessing redox homeostasis and evaluating other proteins or lipids.

Interestingly, in the urine of both ethanol-poisoned subjects and the control group, we observed significantly higher levels of early (kynurenine, N-formylkynurenine, dityrosine, tryptophan) and late (AGE) protein glycation products compared to blood. Although urine is a plasma filtrate containing nearly all metabolic end products [39], previous studies have shown that oxidized and glycated biomolecules are relatively higher in blood than in urine, and only the oxidative DNA damage product (8-hydroxydeoxyguanosine) is significantly greater in urine [40]. Thus, our results indicate increased stability of urine collected post mortem compared to blood samples.

The only significant differences in the redox profile between the study and control groups were found in biomarkers characterizing total antioxidant capacity. We showed significantly higher urinary TAC and FRAP in people who died from alcohol overdose than in sudden death people. It is well known that TAC and FRAP describe the resultant radical scavenging capacity of the analyzed sample and thus provide more information than the evaluation of individual antioxidants separately [41,42]. Nevertheless, 70% of the urine’s antioxidant power depends on uric acid [43]. Although we did not evaluate UA concentrations in the study, the increase in TAC and FRAP levels may be due to hyperuricemia in alcohol drinkers [44]. Indeed, there is an increase in blood UA in people who regularly consume alcohol, resulting in the development of gout manifested by joint inflammation, mainly in the lower extremities [45]. However, even with one-time alcohol abuse, toxic kidney damage/failure may occur, manifesting as an increase in circulating UA [46]. It should be noted that nitrogenous metabolic products (UA) are much less sensitive to autolytic degradation than other redox biomarkers, especially thiol antioxidants/enzymes and biomolecule oxidation products [43,47].

Moreover, uric acid has dual properties [48,49]. UA is a low molecular weight antioxidant at physiological concentrations, but it exhibits pro-oxidant solid properties under hyperuricemia by activating the superoxide generation system via NADPH oxidases [50]. UA also directly stimulates mononuclear cells to produce proinflammatory cytokines such as interleukin 1b (IL-1b, interleukin 1b), interleukin 6 (IL-6, interleukin 6), and tumor necrosis factor-a (TNF-a), which further enhances ROS production [51,52]. Therefore, the increase in urinary TAC and FRAP levels of alcohol-intoxicated subjects may indicate an intensification in oxidative processes. The adaptive response to ROS overproduction in these patients may be increased urinary GPx activity [53].

Exposure to xenobiotics that affect redox homeostasis is a complex phenomenon. At the onset of exposure, we usually observe an adaptive response of the body to strengthen the antioxidant barrier. Therefore, oxidative damage sustained during this period can later be undone by repair mechanisms. Long-term exposure to pro-oxidant substances, can lead to the depletion of antioxidant reserves, which is the cause of chronic oxidative stress and oxidative damage to the body [54,55]. It should be noted that the oxido-reductive balance undergoes continuous and dynamic changes. Assessment of redox biomarkers allows us to capture these changes, even with short-term exposure to oxidative stress modulators [54,55,56,57]. Indeed, alcohol’s effect on the redox balance can be detected in vivo as early as 90 min after administration [58]. Toxic effects of alcohol in in vitro models are observed after only a few minutes, which is also highly dose dependent [59]. Nevertheless, it should be remembered that redox imbalances can already occur at the level of individual cells or subcellular spaces, without modifying the total redox state of the cell or organism [54,55]. Therefore, the assessment of circulating redox biomarkers may be of limited diagnostic value. This is indicated by the results of our study, although further experiments in both animal and human models are required. 

**Figure 5:**
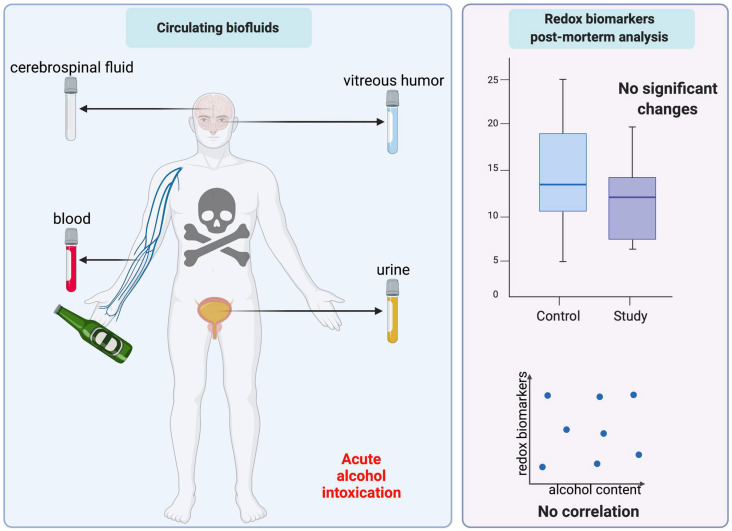
Graphical conclusions from the study.

Summarizing, this paper is the first to compare redox biomarkers in different biological fluids of alcohol-poisoned subjects. We have shown that post-mortem estimation of oxidative stress may have limited diagnostic value in bioliquids, such as blood, urine, vitreous, and cerebrospinal fluid (Figure 5). Nevertheless, the diagnostic material most resistant to post-mortem decomposition was urine. 

## 4. Limitations, Strengths and Future Prospects

We recognize that our study has numerous limitations. Although the body was kept in cold storage until the autopsy, according to Polish law, the autopsy cannot take place earlier than 12 h after the confirmation of death. This time may be reduced only if tissues or organs are needed for transplantation; therefore, we could not collect material for the study earlier [60]. This poses a significant problem in forensic medicine. Nevertheless, in all patients, the material was taken at the same time and processed in the same way. 

Due to the relatively small number of respondents, we realize that our survey is a pilot study. However, the sample size is similar to other forensic studies. We stratified the results with respect to ethanol concentration, cause/circumstance of death, and time of death, which is crucial for post-mortem analysis. Using regression analysis, we unequivocally detected that redox biomarkers did not depend on sex and age (the age of subjects in both groups did not differ significantly, and only middle-aged adults were eligible for the study).

At this point, it is important to emphasize the incredible strengths of the study. An undoubted advantage is the carefully selected study and control group, which did not include people with systemic diseases of oxidative stress etiology. Therefore, a reduced number of subjects were enrolled in the study. We also evaluated an entire panel of redox biomarkers (e.g., enzymatic and non-enzymatic antioxidant defense, total redox status, biomarkers of oxidative, carbonyl, and nitrosative stress), which allows an objective assessment of the oxidation–reduction balance in the subjects. We also assessed redox homeostasis in various biological fluids: blood, urine, vitreous humor, and cerebrospinal fluid. Additionally, this is the first paper to demonstrate the limited diagnostic value of circulating redox biomarkers in individuals collapsed from alcohol intoxication. Thus, our work indicates the need for continued research evaluating the diagnostic utility of circulating bioliquids collected post mortem. Since redox biomarkers are increasingly used in laboratory medicine, our study has both scientific and diagnostic value.

What is the next step? Further studies are needed to assess the intensity of oxidative and carbonyl stress in ethanol-damaged organs and the effects of post-mortem processes on cellular redox balance. In addition, it will be interesting to compare redox biomarkers between those who died of alcohol poisoning and chronic addicts. The evaluation of alternative diagnostic biomaterials, such as saliva, hair, or sweat, to assess redox homeostasis is also indicated. 

## 5. Materials and Methods

The research was approved by the Local Ethical Committee of the Medical University of Bialystok (act no.: R-I-002/82/2013). All methods were performed in accordance with the relevant guidelines and regulations, including the Declaration of Helsinki. Informed consent was obtained from the LAR/guardians of the deceased person.

The research was conducted on two groups of the deceased. The first one (study group) consisted of 22 people (20 males, 2 females; the average age 46 years) who died due to acute ethanol intoxication (BAC in the study group: 4 ‰ or above). The family members and the prosecutor’s investigation did not confirm information about the chronic alcohol consumption in the study group. The second group (control group) included 30 sober persons (22 males, 8 females; the average age 54 years). The causes of death in the control group were brain injuries (19 individuals—63.3%) and chest injuries (11 individuals—36.7%). The death occurred directly at the crime scene, without a phase of agony. After declaring death, the bodies from the scene were immediately transported to a morgue. In both groups, illness changes in organs (especially in the kidney and liver) were excluded. Cancers, chronic inflammatory processes, such as rheumatoid arthritis, and other diseases and unhealthy habits (e.g., diabetes, hypertension, and smoking cigarettes) were also excluded. Family members ruled out drug abuse. The body was stored in the cold at 4 degrees Celsius until post-mortem examination.

The material was collected during the medico-legal autopsy, 12 h after death, with a syringe in the amount of 5 mL from a femoral vein (blood), urinary bladder (urine), eye (vitreous humor), and lateral ventricle of the brain (cerebrospinal fluid). Biological samples were centrifuged at 3000× *g* for 20 min at 4 °C. The supernatant was divided, frozen, and stored in Eppendorf tubes at −80 °C until the biological analysis was performed. The ethanol concentration was determined by the gas chromatography method headspace technique (HS-GC-FID). The rest of the biological material was centrifuged at 3000× *g* for 20 min at 4 °C. The supernatant was divided, frozen, and stored in Eppendorf tubes at −80 °C until the biological analysis was performed. A Thermo Electron Corporation Trace, GC Ultra chromatograph, is equipped with an FID detector and headspace TriPlus automatic injector, with a capillary column a ZB-BAC1 (30 m × 0.32 mm ID × 1.8 μm film thickness) and a ZB-BAC2 (30 m × 0.32 mm ID × 1.2 μm film thickness). The following conditions were used: carrier gas—helium 1.8 mL/min., column temperature—40 °C, injector temperature—150 °C, detector temperature—200 °C, sample heating temperature—60 °C, thermoregulation time—5 min. The standard curve for ethanol ranged from 0.2 to 4.0‰ (Figure S1; LOQ—0.2 ‰, LOD—0.05 ‰). Using this method allows the detection of the metabolite of alcohol: acetaldehyde.

### 5.1. Redox Determinations

All reagents for the biochemical assays (unless otherwise specified) were obtained from Sigma-Aldrich, Germany. The absorbance/fluorescence was measured using Infinite M200 PRO Multimode Microplate Reader, Tecan. All determinations were determined in duplicate samples and standardized to 100 mg of total protein. 

### 5.2. Antioxidant Assays

Catalase (CAT) activity was estimated spectrophotometrically according to Aebi [61] by measuring the decomposition rate of hydrogen peroxide, and the absorbance was measured at 240 nm. One unit of CAT activity was defined as the quantity of enzyme that decomposes one mmol hydrogen peroxide per one minute. 

Superoxide dismutase-1 (SOD-1) activity was analyzed spectrophotometrically by measuring the inhibition of adrenaline oxidation to adrenochrome at 480 nm [62]. It was assumed that one unit of SOD-1 activity inhibits the oxidation of adrenaline by 50%.

Glutathione peroxidase (GPx) activity was determined spectrophotometrically, which is based on the reduction of organic peroxides in the presence of NADPH [63]. The absorbance was measured at 340 nm. One unit of GPx activity was assumed to catalyze the oxidation of 1 μmol of NADPH for one minute.

Glutathione reductase (GR) activity was assayed spectrophotometrically by measuring the decrease in NADPH absorbance at 340 nm [64]. One unit of GR activity was defined as that amount of enzyme which catalyzes the oxidation of 1 μmole of NADPH for one minute.

Reduced glutathione (GSH) content was estimated spectrophotometrically based on the reaction with 5,5′-dithiobis-2-nitrobenzoic acid (DTNB) [64]. The absorbance of the resulting complex was measured at 412 nm. 

### 5.3. Redox Status Assays

Total antioxidant capacity (TAC) levels were determined spectrophotometrically according to Erel [65] based on the reaction with 2,2-azinobis-3-ethylbenzothiazoline-6-sulfonic acid radical cation (ABTS^*+^). Changes in the absorbance were measured at 660 nm. TAC levels were calculated from the calibration curve for Trolox (6-hydroxy-2,5,7,8-tetramethylchroman-2-carboxylic acid).

Ferric reducing ability of plasma (FRAP) levels were assayed spectrophotometrically based on the reaction with 2,4,6-tripyridyl-s-triazine (TPTZ) [66]. Changes in the absorbance were measured at 593 nm. FRAP levels were calculated from the calibration curve for FeSO_4_.

### 5.4. Oxidative Damage Assays

The advanced oxidation protein product (AOPP) concentration was analyzed spectrophotometrically by measuring the oxidative capacity of the iodine ion at 340 nm [67]. For AOPP determination, serum samples were diluted 1:50 (*v:v*) in phosphate-buffered saline, pH 7.2 [68].

Advanced glycation end-product (AGE) content was estimated spectrofluorimetrically by measuring AGE-specific fluorescence at 350 nm/440 nm [67]. For AGE determination, serum samples were diluted 1:50 (*v:v*) in phosphate-buffered saline, pH 7.2 [68].

To detect dityrosine, kynurenine, N-formylkynurenine, and tryptophan, blood samples were diluted (1:10, *v:v*) in 0.1 M H_2_SO_4_. Fluorescence at 330/415, 365/480, 325/434, and 95/340 nm was analyzed, and all results were normalized to fluorescence of 0.1 mg/mL quinine sulfate (in 0.1 M H_2_SO_4_) [69,70].

### 5.5. Nitric Oxide Assay

Nitric oxide (NO) level was determined spectrofluorimetrically by measuring its stable decomposition products NO_3_^-^ and NO_2_^-^ by the Griess reaction [71,72]. Changes in the absorbance were measured at 543 nm. 

### 5.6. Protein Assay

Total protein content was estimated using the bicinchoninic acid (BCA) method [49] with the commercial kit Thermo Scientific PIERCE BCA Protein Assay (Rockford, IL, USA).

### 5.7. Statistical Analysis

Statistical analysis was performed using Statistica 12.0 version (StatSoft, Tulsa, OK, USA). Given the lack of a normal distribution, the Mann–Whitney U test and Spearman correlation were used in this study, and the results were presented as a median (minimum–maximum) and percentiles. Multivariate analysis of the simultaneous impacts of many independent variables on one quantitative dependent variable was made using linear regression. Alcohol concentration and cause of death were included as independent variables. The models were further adjusted for gender and age (the reference category is female gender; head injury, chest injury are dummy variables; and the reference category is the cause of death, alcohol).

## Data Availability

The data that support the findings of this study are available from the corresponding author upon reasonable request. Some data may not be made available because of privacy or ethical restrictions.

## Supplementary Materials

The following supporting information can be downloaded at: https://www.mdpi.com/article/10.3390/ijms231911808/s1.
